# Proposing Development and Utility of a Mobility Composite Measure in Patients with a Neurologic Disorder

**DOI:** 10.1155/2017/8619147

**Published:** 2017-10-25

**Authors:** Chad Swank, Sattam Almutairi, Ann Medley

**Affiliations:** ^1^School of Physical Therapy, Texas Woman's University, 5500 Southwestern Medical Ave., Dallas, TX 75235-7299, USA; ^2^Physical Medicine and Rehabilitation, Baylor Institute for Rehabilitation, 909 N. Washington Ave., Dallas, TX 75246, USA

## Abstract

**Background:**

Outcome measures typically assess single domains making holistic assessment difficult. Our purpose was to develop a mobility composite measure (MCM) based on four commonly used outcome measures and compare this composite score to the individual measures in patients with neurologic disorders.

**Methods:**

We retrospectively reviewed 148 medical records for inclusion of primary neurologic diagnosis and scoring for 5 times sit-to-stand test (5TSST), 10-meter walk test (10MWT), 2-minute walk test (2MWT), and activities-specific balance confidence (ABC) scale.

**Results:**

After establishing that a single concept was being assessed with interitem correlations, raw scores were converted to percentage of normal and combined into the MCM for analysis from admission to discharge. Scores on each measure significantly improved after intervention (5TSST, *p* < .001; 10MWT, *p* < .001; 2MWT, *p* < .001; ABC, *p* = .02). Mean MCM (*n* = 93) admission scores were 67.55 ± 31.88% and discharge scores were 74.81 ± 34.39% (*p* = .002). On average, patients improved 7.26% on the MCM exceeding the threshold of expected error (MDC_95_ = 3.59%).

**Conclusions:**

MCM detected change in patient outcomes statistically and clinically and appears to capture a holistic picture of functional status. We recommend a prospective study to further investigate a “composite measure” incorporating measures from several functional domains.

## 1. Introduction

Assessing mobility requires analysis across domains of impairment, activity, and participation. While individuals may subjectively report their level of function, objective measures identify functional problems that individuals or family did not report [1, 2]. Recent emphasis on outcome determination has led to an increased variety of performance measures to assess patients' physical function. However, implementing outcome measures into a clinical setting is often beset by challenges including therapist efficiency and measurement reliability and validity [3]. Further, outcome measures often assess a single construct which may limit therapists' ability to evaluate the impact of therapeutic intervention on the person with deficits across multiple domains. Consequently, therapists utilize multiple outcome measures to adequately assess a person across domains (body function/structure, activity, and participation). Unfortunately, outcome measures are likely to have disparate scales making a holistic assessment of the patient challenging.

Development of a composite measure may mitigate limitations associated with single or multiple measures and capture the concept of system performance. That is, a single tool comprised of multiple domains may be of value to more holistically describe the patient's status. Physical therapists commonly assess physical function domains including the cardiovascular system, integumentary system, musculoskeletal system, neuromuscular system, and attention/cognitive ability [4] in patients with neurologic dysfunction. Each of these physical function domains may contribute to capacity and performance observed in functional mobility. Therefore, development of a single composite tool bridging domains may maximize clinician effort in holistically describing the functional status of a patient.

Two examples in the literature from the mid-1990s describe the development of a composite measure. First, the Short Physical Performance Battery (SPPB) was created to assess functional status in elderly adults [5]. Tests of balance, walking, and lower extremity strength were summed according to categorical performance rankings and able to distinguish risk of mortality and nursing home admission. Second, the National Multiple Sclerosis Society developed a novel outcome measure to assess relevant clinical domains which had desirable characteristics (i.e., relevant, valid, reliable, sensitive, and practical) [6]. The resulting measure, the Multiple Sclerosis Functional Composite (MSFC) measure, was comprised of three previously existing scales (25-foot walk test, Nine-Hole Peg test, and Paced Auditory Serial Addition test) [7]. The MSFC allows the clinician to comprehensively describe the functional status of the individual with MS and monitor improvement with treatment or disease deterioration.

Similarly, we aimed to adopt the aforementioned desirable outcome measure characteristics for creating a “composite measure” allowing for both assessments of single domains and a holistic perspective of a patient's status. However, the SPPB and MSFC were developed for populations of the elderly and people with MS, respectively. We proposed the development of a “composite measure” of existing outcome scales to assess functional domains often impaired in people with neurologic insult. The purpose of our study was to develop a mobility composite measure (MCM) based on four commonly used outcome measures and compare this composite score to the individual measures in patients with neurologic disorders.

## 2. Methods

### 2.1. Procedures

We retrospectively reviewed medical records of patients who attended a hospital based outpatient physical therapy clinic over a period of 28 months. Approval of the local hospital institutional review board was obtained prior to initiating review of the medical records. Medical records of patients with neurologic diagnosis were screened for inclusion criteria. Medical records documenting performance on at least three of the four outcome measures (5 times sit-to-stand test (5TSST), 10-meter walk test (10MWT), 2-minute walk test (2MWT), and activities-specific balance confidence (ABC) scale) of interest during an initial evaluation were included in this study. Patients without a primary neurologic diagnosis were excluded from review.

Medical records were reviewed by two individuals trained to extract data. Initial training included a review of the variables to be collected, the procedure manual, and the data entry form. Following training, the data extractors were each given two medical records for practice. The research team audited the extracted data for accuracy. Continuous oversight of data extraction was provided throughout the medical record review process by the research team. Data extracted from medical records that met inclusion criteria included age, gender, number of visits, payor source, diagnoses and diagnostic categories, assistive device, and performance measures during initial evaluation, reevaluation, and discharge sessions.

### 2.2. Outcome Measures

In this study we selected four outcome measures to be included in our proposed MCM: 5TSST, 10MWT, 2MWT, and ABC. These measurements were chosen because they are routinely conducted in our setting. They also assess distinct components of physical function including lower extremity strength, gait speed, endurance, and balance confidence. Of note, each measure is recommended for clinical use across the continuum of care by the NeuroEDGE Task Force of the Neurology Section of the American Physical Therapy Association [13].

#### 2.2.1. Five Times Sit-to-Stand Test (5TSST)

The 5TSST was used to assess lower extremity strength and balance and is an indicator of postural control [14]. This tool is also used to measure change following intervention [15]. The 5TSST is used in a variety of diagnoses including people with arthritis [14], renal disease [16], stroke [17], older adults [8], and Parkinson's disease [18]. Sitting on a chair of 45 cm seat-to-floor height with arms folded across chest, the patients are instructed to stand up fully and sit down five times as quickly as they can. Timing, recorded by stopwatch, begins at “go” and stops when the patient's buttocks touch the chair on the last repetition. For the community-dwelling elderly, a cut-off score of greater than 15 seconds places them in a category of risk for falls [19]. Normal scores for individuals aged 60 to 80 years range from 11.4 to 12.7 seconds [8]. A minimal detectable change (MDC_95_) of 2.3 seconds has been identified in patients with Parkinson's disease [20]. This test has demonstrated excellent test-retest reliability (ICC = .957) and appears to have concurrent validity with self-reported physical function in community-dwelling adults [21].

#### 2.2.2. Ten-Meter Walk Test (10MWT)

Gait speed was used to assess walking function and predict health status and functional limitations [22]. While several walking measures have been suggested for assessment of walking function, we selected the 10MWT because it assesses self-selected preferred walking speed with or without an assistive device, is responsive to change (effect size = 1.17; standardized response mean = 1.68 with good inter- and intrarater reliability) [23], and is widely used in neurological patients [24]. Individuals are asked to walk a 14-meter path which includes a 2-meter acceleration zone, a 10-meter measurement zone, and a 2-meter deceleration zone. Speed is calculated for the 10-meter distance between the acceleration and deceleration zones. Normal gait speed for adults older than 50 years is >1.27 m/s [10]. Further, functional mobility can be categorized based on gait speed: household ambulators (<0.4 m/s), limited community ambulators (0.4 to 0.8 m/s), and community ambulators (>0.8 m/s) [25]. The MDC_95_ of 0.13 m/s indicates substantial meaningful change [9].

#### 2.2.3. Two-Minute Walk Test (2MWT)

Cardiovascular function is critical to improving quality of life and increasing physical and emotional participation in everyday activities [26]. The 2MWT demonstrated reliability and validity for the assessment of cardiovascular capacity in patients with moderate-to-severe COPD [27] and elderly individuals [28]. During the 2MWT the patient is asked to walk for two minutes at the fastest speed possible with or without an assistive device. The mean distance for community-dwelling older adults is 150.4 meters with an MDC_90_ of 12.2 meters [11]. Test-retest reliability has been established as excellent (ICC = 0.97) for individuals with neurologic dysfunction [29].

#### 2.2.4. Activities-Specific Balance Confidence (ABC) Scale

As a standardized self-report measure, the ABC asks participants to rate their balance confidence when completing specific daily life balance challenges. The ABC consists of 16 items describing various activities for which participants are asked to rate their confidence in maintaining balance on a scale of 0% (not confident) to 100% (completely confident) and may be administered as an interview. Final scores are determined by calculating the average score on the 16 items. In addition, the ABC provides the clinician insight into the patient's cognitive function while assisting clinicians to target appropriate interventions [30]. The ABC accurately distinguishes between fallers and nonfallers and has adequate sensitivity (89%) and specificity (96%) [12, 31]. The ABC also demonstrates excellent test-retest reliability (*r* = .92) [30].

### 2.3. Data Analysis

Statistical analysis was completed using IBM SPSS Statistics 19. Descriptive statistics were used to summarize patient characteristics. We performed separate paired *t*-tests to compare differences between admission and discharge scores on each of the four outcome measures (5TSST, 10MWT, 2MWT, and ABC). Times recorded during the 10MWT were converted to gait speed (meters/second) for analysis. Type I error was set at 0.05.

Before a composite score was calculated, the baseline outcome measures were tested with interitem correlations to determine if a single concept (i.e., mobility) was being assessed. Interitem correlations provide an assessment of the extent to which variables assess the same concept [32]. Correlations lower than 0.20 indicate that the variables may not be representative of an overarching domain.

To calculate a clinically relevant composite score, raw scores were standardized for each of the measures at all time points. In development of the MSFC, *z*-scores were computed for each individual measure based on established population means and then combined into a single composite score [33]. However, for our study, the database was not large enough to develop *z*-scores. Therefore, to enable comparison across different scales, we transformed raw scores into percentage [34] calculations based upon established reference normal values for each measure at each time interval (Table 1). Once they are converted to percentage of normal values, we calculated the MCM score using the equations listed in Table 2. For the 5TSST, we used the 12.0 seconds (mean difference between 11.4 seconds and 12.6 seconds) as the reference value. In order to accommodate direction of the percentage scores where closer to 100% equals closer to normal function, we used the inverse of 5TSST. The reference normal score used for each outcome measure was not intended to imply a maximal score. As such, it was possible to attain a score greater than 100% on each outcome measure. For instance, the reference normal score for gait velocity was 1.27 m/s and a patient who walked at this velocity would receive a 100% reference normal score. However, it is possible for some patients to walk at a preferred velocity greater than 1.27 m/s. For these individuals, they would receive scores greater than 100%.

As anticipated in a retrospective medical record review, we observed missing outcome measure data in the medical record. We calculated a composite score for each patient only if admission and discharge outcome measure scores were available on at least three of the measures (5TSST, 10MWT, 2MWT, and ABC). Using a complete-case analysis approach to missing data, we excluded cases from analysis when two or more outcome measures were missing as being initially or terminally assessed in the medical record. Missing values were not replaced.

## 3. Results

A total of 169 patient records were initially reviewed. After screening, 21 records were excluded. The remaining 148 records were included for analysis. The mean age of the patients was 63.12 ± 17.19 years, 54.73% were men (*n* = 81), and mean number of physical therapy visits was 11.28 ± 11.51. Details for patient diagnoses included in analysis are listed in Table 3. The majority of patients (75%) had acquired adult disease or a progressive neurologic disorder.

Paired *t*-test analysis of the raw scores (i.e., before conversion to percentage) showed significant improvement from admission to discharge for each individual outcome measure (Table 4). Further, the mean change exceeded the MDC values for individual outcome measure (Table 1). Consequently, the mean scores reflect improved functional lower extremity strength (5TSST), lowered risk of falls (5TSST, ABC), increased gait speed (10MWT), and gait capacity and endurance (2MWT). As importantly, our patients moved categorically from being limited community ambulators to community ambulators [22].

Interitem correlations provide an assessment of the extent to which variables assess the same concept [32]. As shown in Table 5, the outcome measures all have interitem correlations higher than .20, indicating enough relatedness to warrant creating a composite score.

Table 6 details the standardized mean percent values for each measure. By including only those patients for whom the medical record contained admission and discharge outcome measure scores for at least three of the measures, we calculated the MCM with a sample size of 93. Calculation of the MCM yielded an admission score of 67.55 ± 31.88% and a discharge score of 74.81 ± 34.39%. That is, the patients overall mobility status was about 68% of normal at admission to physical therapy and significantly improved to nearly 75% of normal at discharge (*p* = .002). Using the formula MDC_95_ = 1.96 × SEM × √2, the MDC_95_ was 3.59% for the MCM. On average, our patients demonstrated an improvement of 7.26% exceeding the threshold of expected error. However, the percentage of participants who exceeded the MDC_95_ on the composite score was only 32.3%. The percentage of individuals demonstrating holistic improvement on the MCM was considerably less than the percentage of patients who exceeded the MDC_95_ on domain specific measures: 5TSST (66.7%), ABC (59.1%), 10MWT (54.8%), and 2MWT (50.0%).

The variation in patient sample size for each analysis was a direct reflection of the missing data from our retrospective medical chart review. Not all objective measures were collected (or at least recorded in the medical record) for all patients at all time points (admission and discharge). For the paired *t*-test analysis, only patients with both admission and discharge scores were included in the analysis (Table 4). However, all objective measure scores recorded in the medical record were converted to percentage of reference normal values (reflected in Table 6).

## 4. Discussion

The purpose of this project was to propose the development of a composite measure as a way to holistically describe a person with neurologic dysfunction. The “mobility composite measure (MCM),” tailored to assess the functional domains often limited in neurological patients, may be helpful to monitoring the course of recovery compared to a normal population. By incorporating recommended, simple, and commonly utilized outcome measures (5TSST, 10MWT, 2MWT, and ABC) the intent of the MCM is to help clinicians make comprehensive decisions regarding the functional status of the patient.

In this retrospective study, we observed improvement on each outcome measure (5TSST, 10MWT, 2MWT, and ABC) from admission to discharge indicating improvement in lower limb strength, endurance, balance confidence, gait velocity, and community ambulatory function. Collectively, standard of care physical therapy appears to have resulted in improvements in separate functional performance measures. The MCM captured this functional improvement but also added value as a global and holistic status measure in comparison to healthy community-dwelling adults. Incorporating the MCM, or the concept of a “composite measure,” into clinical practice may provide several advantages for clinicians, physicians, and insurers. Firstly, it allows for individual measurement of commonly limited functional domains in patients with neurological disorder patients while contextualizing each measure within the overall patient function. Specifically, the MCM assesses functional activities across musculoskeletal, neuromuscular, cardiovascular, and cognitive system domains. Though we observed a mean improvement on each scale, a common clinical phenomenon is for a patient to improve in some, but not all, assessed domains. Our results expressed by the percentage of patients who exceeded the MDC_95_ values underscore this clinical phenomenon. Specifically, only one-third of patients demonstrated improvement on the MCM while improvement in individual domain scales was observed more than 50% of the time. In this clinical scenario, the therapist faces a dilemma of attempting to describe to the patient, physician, and insurer the overall patient status. By combining the clinical measurement scores into a single composite score, the relative progress or regress observed on each individual measure is inherently included. That is, the MCM places both patient progress and regress on individual measures within the context of the function of the whole person.

A second way the MCM adds value is by allowing the clinician opportunity to holistically describe functional improvement and allowing comparison to healthy community-dwelling adults. By transforming the individual scales into a percentage (as opposed to a *z*-score), the MCM may be more user-friendly and easier to interpret for physical therapy clinicians, physicians, and insurance providers. Whether a diagnosis of progressive disease (i.e., multiple sclerosis, Parkinson's disease) or acute onset (i.e., stroke, brain injury), the ability to holistically monitor regression from or recovery towards health may be of value to healthcare professionals. Lastly, the potential value of our holistic MCM percentage is not offset by additional therapist burden. Calculation of the MCM is relatively simple using a basic calculator and is minimally time-consuming.

Our study has obvious limitations. First, this study is a retrospective design limiting our control over what data was collected and reported resulting in missing data. While there are potentially many reasons for why data are missing, particularly in retrospective studies, one reason is that a patient may not be able to complete the test. For example, a patient may not be able to complete 5 sit-to-stand trials. Accordingly, no score (a missing value) would be recorded for this patient. For future “mobility composite measure” tests it may be of value to construct them such that a meaningful zero can be obtained rather than a missing value. For example, rather than 5 sit to stands, the number of sit to stands in 30 seconds may be an option. If a patient cannot complete one sit to stand, a score of zero would be assigned and contribute to the analysis. Second, we were unable to mimic the MSFC due to a limited sample size and the absence of a large dataset from which to determine *z*-scores for each patient. However, comparing our patients to normal through the use of a percentage may be more easily understood by the clinician enabling its adoption into the clinic setting. A corollary limitation of our small heterogeneous sample size is the rather large variance observed in our statistical calculations (i.e., standard deviation). As such, caution is recommended while interpreting our results. A third limitation is that our model considers each measure as equally valuable in describing a patient's functional status. It may be that one measure (i.e., gait speed) may be more important than another (i.e., self-perceived balance confidence) in holistically determining a patient's status. We recommend that future studies with a prospective design should determine appropriate weighting to our composite measure.

## 5. Conclusion

In this retrospective study, we developed a composite measure to holistically describe function of patients with deficits in multiple system domains due to neurological dysfunction. Though the observed improvement on our MCM was statistically significant and exceeded expected error, future studies will be necessary to determine if this change was clinically important. Nonetheless, the adoption of a “mobility composite measure” for patients with neurologic disorders may provide clinical value.

## Figures and Tables

**Table 1:** Selected outcome measures and related domains.

Measure	ICF domain	System domain	Application	MDC	Norms
5TSST	Impairment/activity	Musculoskeletal	Functional strength, risk of falls	2.3 s [8]	11.4–12.6 s [8]
10MWT	Activity	Neuromuscular	Gait speed, functional ambulatory classification	0.13 m/s [9]	>1.27 m/s [10]
2MWT	Activity	Cardiovascular	Endurance, gait capacity, gait speed	12.2 m [11]	150.4 m [11]
ABC scale	Activity/participation	Attention/cognition	Self-perception of balance, risk of falls	13%	<67% fall risk; >80% normal [12]

*Note*. ICF, international classification of functioning, disability and health; MDC, minimal detectable change; 5TSST, 5 times sit-to-stand test; 10MWT, 10-meter walk test; 2MWT, 2-minute walk test; ABC scale, activities-specific balance confidence scale.

**Table 2:** Conversion of equations to percentage.

Scale	Equation
5TSST	$\left[\frac{1}{\left(X\;\;seconds/12.0\;\;seconds\right)}\right]\times 100$
10MWT gait velocity	$\left(\frac{X\;\text{m/s}}{1.27\;\text{m/s}}\right)\times 100$
2MWT	$\left(\frac{X\;\;\text{meters}}{150.4\;\;\text{meters}}\right)\times 100$
ABC	$\left(\frac{X\%}{80\%}\right)\times 100$
MCM	$\frac{\left(ABC\%+5TSST\%+2MWT\%+Gait\;\;Velocity\%\right)}{4}$

*Note*. 5TSST, 5 times sit-to-stand test; 10MWT, 10-meter walk test; 2MWT, 2-minute walk test; ABC scale, activities-specific balance confidence scale; MCM, mobility composite measure; *X* = raw score achieved by patient on each scale.

**Table 3:** Participant diagnostic categories.

Diagnostic categories	Number of patients
Vestibular disorders	13
Acquired adult disease (i.e., CVA, TBI)	68
Progressive disease (i.e., MS, PD)	44
Neuropathy	13
Spinal cord injury	10
Total	148

*Note*. CVA: cerebrovascular accident; TBI: traumatic brain injury; MS: multiple sclerosis; PD: Parkinson's disease.

**Table 4:** Performance measure comparison of baseline to discharge (raw scores).

Performance measures	Baseline	Discharge	*p* value	*N*
	Mean (SD)	Mean (SD)		
5TSST (s)	22.07 (13.13)	15.70 (14.71)	<0.001	66
10MWT gait speed (m/s)	0.73 (0.43)	0.91 (0.46)	<0.001	73
2MWT (m)	76.55 (39.28)	96.19 (45.68)	<0.001	50
ABC scale (%)	46.12 (23.39)	60.95 (27.68)	0.02	22

*Note*. Paired *t*-test analysis; SD, standard deviation; 5TSST, 5 times sit-to-stand test; 10MWT, 10-meter walk test; 2MWT, 2-minute walk test; ABC scale, activities-specific balance confidence scale; raw scores are actual recorded scores obtained from the medical record prior to conversion to percentage of reference normal value.

**Table 5:** Interitem correlations between outcome measures.

Variable	5TSST	10MWT velocity	2MWT	ABC scale
5TSST	—			
10MWT gait speed	−0.647	—		
2MWT	−0.630	0.848	—	
ABC scale	−0.433	0.511	0.488	—

*Note*. 5TSST, 5 times sit-to-stand test; 10MWT, 10-meter walk test; 2MWT, 2-minute walk test; ABC scale, activities-specific balance confidence scale.

**Table 6:** Performance measure comparison of baseline to discharge (percentage of normal).

Performance measures	Baseline	*N*	Discharge	*N*	Δ
	Mean (SD)		Mean (SD)		
5TSST (%)	81.20 (50.37)	116	100.12 (46.96)	70	18.92
10MWT (%)	63.15 (34.80)	115	71.53 (34.75)	80	8.38
2MWT (%)	53.78 (28.63)	86	62.21 (28.96)	61	8.43
ABC scale (%)	59.22 (34.31)	61	72.37 (35.86)	25	13.15

*Note*. SD, standard deviation; 5TSST, 5 times sit-to-stand test; 10MWT, 10-meter walk test; 2MWT, 2-minute walk test; ABC scale, activities-specific balance confidence scale.
